# Isolation and Characterization of a *Klebsiella* Phage H33IIK Targeting Multidrug-Resistant *Klebsiella pneumoniae* in Peru

**DOI:** 10.3390/antibiotics15040365

**Published:** 2026-04-01

**Authors:** Elsa Aguilar-Ancori, Marishani Marin-Carrasco, Duly Nuñez-Carazas, Abraham Espinoza-Culupú, Pablo Ramirez, Enrique Mamani-Zapana

**Affiliations:** 1 Laboratory of Microbiology and Immunology, Biological Science Faculty, Universidad Nacional de San Antonio Abad del Cusco, Cusco 08003, Peru; shmarin243@gmail.com (M.M.-C.); duly.nunez@unsaac.edu.pe (D.N.-C.); 2 Laboratory of Molecular Microbiology and Biotechnology, Biological Science Faculty, Universidad Nacional Mayor de San Marcos, Lima 15081, Peru; aespinozac@unmsm.edu.pe (A.E.-C.); pramirezr@unmsm.edu.pe (P.R.); 3 Laboratory of Virology and Clinical Molecular, Biological Science Faculty, Universidad Nacional Mayor de San Marcos, Lima 15081, Peru; emamaniz@unmsm.edu.pe

**Keywords:** multidrug-resistant, *Taipeivirus*, bacteriophages, lytic activity

## Abstract

**Background**: The global rise in multidrug-resistant (MDR) Gram-negative bacteria (GNB) poses an urgent challenge for infection control and antibiotic stewardship. Among these, *Klebsiella pneumoniae* is a major cause of hospital-acquired infections and is listed as a critical priority pathogen by the World Health Organization. Peru reports an exceptionally high prevalence of MDR *K. pneumoniae*, underscoring the need for innovative antimicrobial approaches. **Methods**: Here, we describe the isolation and characterization of lytic *Klebsiella* bacteriophage from sewage samples collected from the Huatanay River (Cusco, Peru) in 2023. Phages were isolated using the reference strain MDR *K. pneumoniae* ATCC BAA-2814. Then, they were screened against 50 clinical MDR *K. pneumoniae* strains. **Results**: The phage H33IIK demonstrated effective antibacterial capability, showing strict host specificity for *K. pneumoniae*, thermal stability, moderate pH tolerance, and high burst size. Whole-genome sequencing analysis classified it within the class Caudoviricetes, family *Ackermannviridae*, and genus *Taipeivirus*. The genomic analysis confirmed the absence of lysogeny-related, antimicrobial resistance, and virulence genes, supporting its suitability and safety for potential biotechnological applications. **Conclusions**: These findings highlight phage H33IIK as a lytic agent effective against MDR *K. pneumoniae*. It could contribute to the development of phage-based approaches to combat MDR GNB.

## 1. Introduction

Multidrug-resistant *Klebsiella pneumoniae* is an agent responsible for nosocomial infections with high morbidity and mortality. This is due to the ease of horizontal transfer of mobile genetic elements such as plasmids, which confer resistance genes and virulence factors [1,2,3,4]. In this context, global health policies have focused on reducing antimicrobial resistance and searching for therapeutic options [5,6]. Thus, bacteriophage therapy represents a promising alternative to address infections caused by MDR *K. pneumoniae*, highlighting the need to identify and characterize effective bacteriophages for clinical use [7].

Bacteriophages, or viruses that infect bacteria, are present in the biosphere [8]. They have a density approximately ten times greater than the current population of bacterial cells. They can be found in various environments, including seawater, sediments, soils, deserts, human and animal microbiomes, rivers, and wastewater [9,10]. Consequently, they are relatively accessible for isolation [11], have great potential to combat infections caused by MDR *K. pneumoniae* [3,12,13], and are also used as nutritional supplements [14]. Lytic bacteriophages possess characteristics that enable them to eliminate or reduce the bacterial load [15]. They are characterized by their ability to target and eliminate a specific bacterial species or a related subgroup [15,16,17,18].

The accumulation and replication of the viral pathogen at the infection site characterize the initial phase of the infection. They encode enzymes that degrade biofilms, particularly those linked to challenging infections caused by hypervirulent and multidrug-resistant *K. pneumoniae* [11]. Studies indicate their potential in combined antibiotic-bacteriophage cocktails, effectively targeting pathogens [11,19] and acting as predators par excellence of these bacteria [17].

Several studies have successfully isolated bacteriophages with lytic activity against multidrug-resistant and hypervirulent *K. pneumoniae*. For example, phage vB_KpnM-20 encodes capsular depolymerases specific for virulent *K. pneumoniae* strains [20]. Similarly, Domingo et al. (2020) [21] described a phage with capsular activity targeting *K. pneumoniae* type K22, which encodes putative depolymerases capable of degrading the capsules corresponding to this serotype. Furthermore, phage VTCCBPA43 demonstrated tolerance to a wide range of temperatures and pH, and its inoculation into a murine pneumonia model significantly reduced the severity of lesions, suggesting a beneficial therapeutic effect [22]. Likewise, phages of the *Taipeivirus* genus have been identified with low intergenomic similarity to previously described phages, which exhibit marked antibiofilm activity against different strains of *K. pneumoniae* [23]. Finally, a recent study indicates that viruses of the *Taipeivirus* genus have a restricted host range but are capable of inhibiting the growth of hypervirulent strains of *K. pneumoniae* [24].

In view of this potential, the present study aimed to isolate and comprehensively characterize a novel lytic bacteriophage targeting MDR *K. pneumoniae*. To achieve this, we employed a systematic methodological approach. First, bacteriophages were isolated from environmental wastewater samples using the reference strain MDR *K. pneumoniae* ATCC BAA-2814 as the host. Following isolation, the host range of the phage was determined by screening its lytic activity against a panel of 50 clinical MDR *K. pneumoniae* strains, as well as against other clinically relevant Gram-negative bacteria. Key phenotypic characteristics, including infection kinetics (optimal multiplicity of infection, one-step growth curve, and killing curve), were then elucidated to assess its replication efficiency and antibacterial potency. Furthermore, its physicochemical stability was evaluated under various thermal, pH, and chemical conditions. Finally, a comprehensive in silico characterization was performed through whole-genome sequencing, followed by phylogenetic analysis and functional annotation to determine its taxonomic position, genomic architecture, and the presence of any genes related to lysogeny, virulence, or antimicrobial resistance. This integrated approach provides a complete biological profile of the isolated phage, from its environmental source to its genomic blueprint, thereby assessing its potential suitability for therapeutic applications.

## 2. Results

### 2.1. Geographic Distribution of the Sample Points

**Figure 1:**
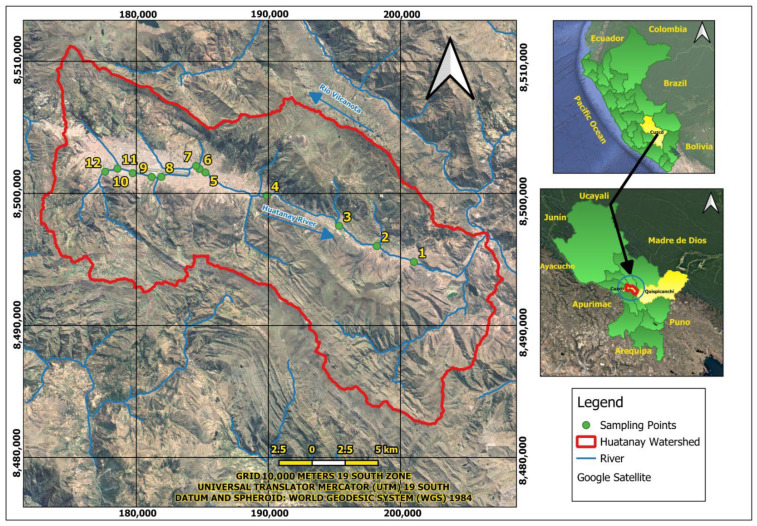
Wastewater sampling points for the isolation of bacteriophage H33IIK; the wastewater treatment plant is at point 4.

Twelve wastewater sampling points were identified, and 72 wastewater samples were obtained at 3300 and 3400 m above sea level; the sampling distribution can be observed in Figure 1.

### 2.2. Isolation and Purification, Host Range, Optimal Multiplicity of Infection, Growth Curve, and Killing Curve

**Figure 2:**
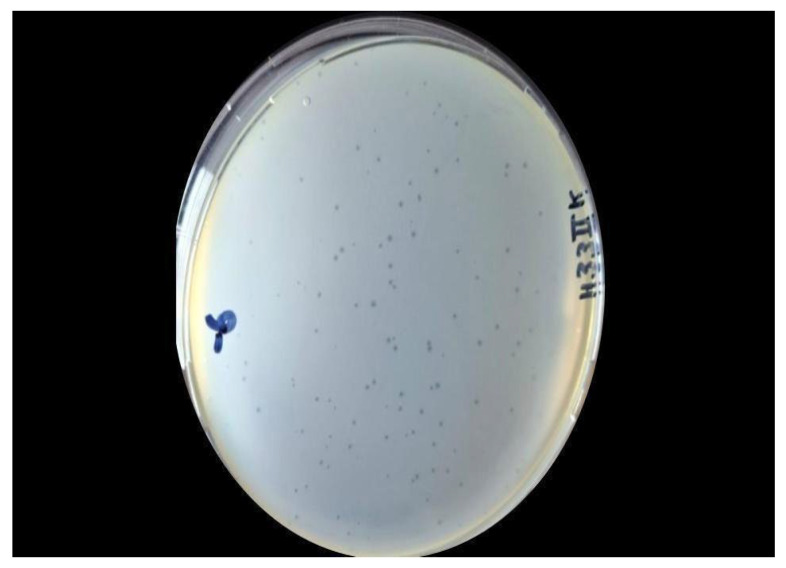
Plaques of bacteriophage H33IIK at 10^−6^ dilution using double-layer agar technique.

Phage H33IIK exhibited lytic activity against *K. pneumoniae* strains producing extended-spectrum β-lactamases (ESBL) and carbapenemases, as observed in Figure 2.

The phage H33IIK was also tested against a panel of 50 MDR *K. pneumoniae* clinical strains, as shown in Table 1, where only the positive lytic activity of the bacteriophage is observed.

The host range was determined by challenging strains of *Enterobacter cloacae*, *Proteus* spp., *E. coli*, and *Pseudomonas aeruginosa.* It was observed that phage H33IIK was exclusive to *K. pneumoniae*, as shown in Table 2.

**Figure 3:**
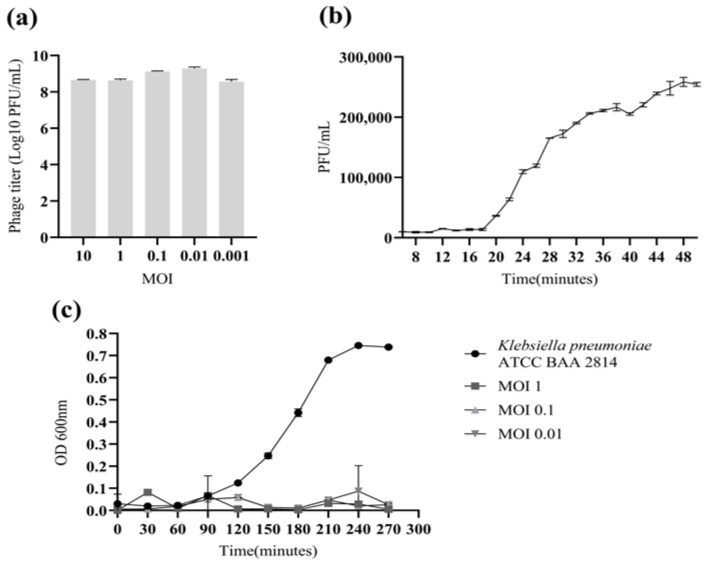
(**a**): The optimal multiplicity of infection (MOI); (**b**): the growth curve of the H33IIK bacteriophage; (**c**): killing curve.

Firstly, the bacterial culture was incubated for six hours until reaching a concentration of approximately OD_600_(0.4–0.5). Subsequently, 1 mL of phage H33IIK (10^5^ PFU/mL) was mixed with 1 mL of the bacterial suspension in TSB tubes at varying bacterial concentrations (10^4^–10^8^ CFU/mL). Then, it was incubated for 6 h, and the concentrations were counted afterwards by double-layer selection. The optimal multiplicity of infection (MOI) for H33IIK was 0.1. Secondly, the one-step growth curve of bacteriophage H33IIK presents a latency period of 20 min and a burst size of 73 PFU/cell (see Figure 3), as shown in the Supplementary Materials.

A killing curve assay was performed by incubating the bacterial host with bacteriophage H33IIK at different multiplicities of infection (MOIs: 1, 0.1, and 0.001). After 270 min of incubation, the phage-free control culture reached an OD_600_ of 0.73 ± 0.05, and reached OD_600_ = 0.005 ± 0.002 at MOI 1); MOIs of 0.1 and 0.001 reached OD_600_ values of 0.026 ± 0.004, respectively.

### 2.3. Thermostability and pH Sensitivity of the Phage and Chloroform Assay

**Figure 4:**
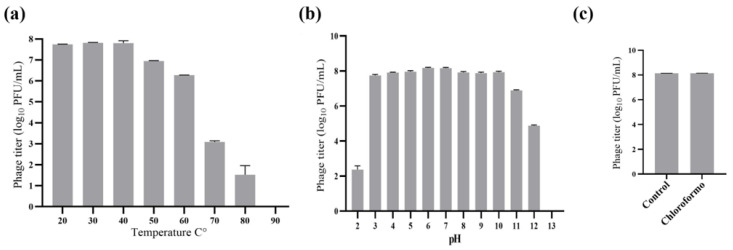
(**a**) Temperature tolerance assay, (**b**) pH sensitivity assay, and (**c**) chloroform sensitivity.

The thermostability of phage H33IIK was evaluated across a temperature gradient over a one-hour exposure period. The phage remained stable between 20 °C and 40 °C, while a significant reduction in phage counts was observed at temperatures above 50 °C (Figure 4a). The pH stability assay showed that H33IIK maintained stability within a pH range of 3.0–10.0. In contrast, exposure to highly acidic (pH 2.0–3.0) or strongly alkaline (pH 11.0–13.0) conditions resulted in a marked decrease in phage numbers (Figure 4b). Finally, exposure to chloroform for one hour demonstrated that H33IIK is resistant to organic solvent treatment, as no significant differences in viral titers were observed compared with the untreated control (Figure 4c).

### 2.4. In Silico Genome Characterization of Phage H33IIK

**Figure 5:**
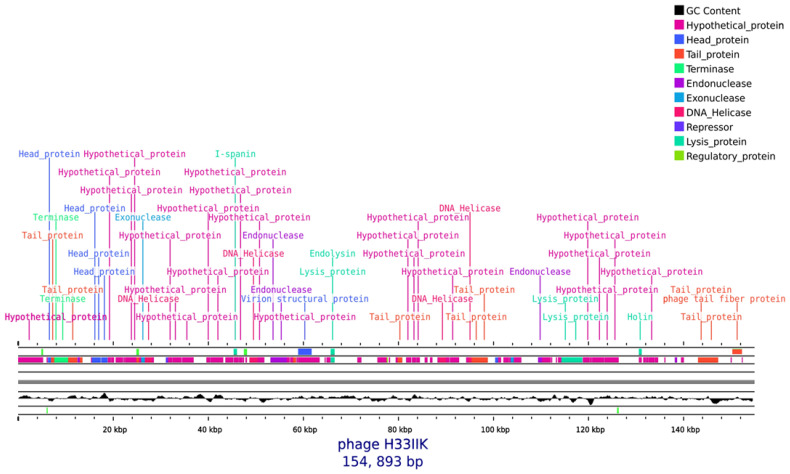
Genome map of bacteriophage H33IIK. The linear genome organization of phage H33IIK is shown, displaying annotated coding sequences (CDSs) on both strands. ORFs are color-coded according to predicted functional categories, including structural proteins (head and tail), DNA packaging proteins (terminase), DNA metabolism enzymes (DNA helicase, endonuclease, exonuclease), and lysis-associated proteins (holin, endolysin, spanins). Additional annotations include regulatory proteins and numerous hypothetical proteins, which constitute the majority of the genome and suggest the presence of uncharacterized functions. The inner track represents GC content variation across the genome. This modular organization is characteristic of tailed double-stranded DNA bacteriophages.

The genome of phage H33IIK consists of a linear, double-stranded DNA molecule of 154,893 bp with a GC content of 44.6%. Analysis using Pharokka and Proksee revealed 200 coding sequences (CDS) and six tRNA genes distributed throughout the genome Figure 5. The genes are organized into functional modules typical of *Taipeivirus* members, including structural proteins of the virion (major capsid, portal, terminase, baseplate, and tail fibers), enzymes involved in DNA replication and repair (polymerase, helicase, topoisomerase, exonuclease, and ligase), as well as lysis-related proteins and others linked to auxiliary metabolic functions. Six tRNA genes (Gln, Tyr, Asn, Ser, Arg, and Met) were also identified, which may help optimize translation efficiency during infection in *K. pneumoniae*.

The in silico analysis did not reveal genes associated with antimicrobial resistance, virulence, or integrases, suggesting hypothetically a lytic lifestyle and a potentially safe profile for phage H33IIK. Overall, the genome exhibits a modular organization typical of the *Taipeivirus* genus, with a predominance of structural and replication-related genes.

### 2.5. Phylogenomic Analysis

**Figure 6:**
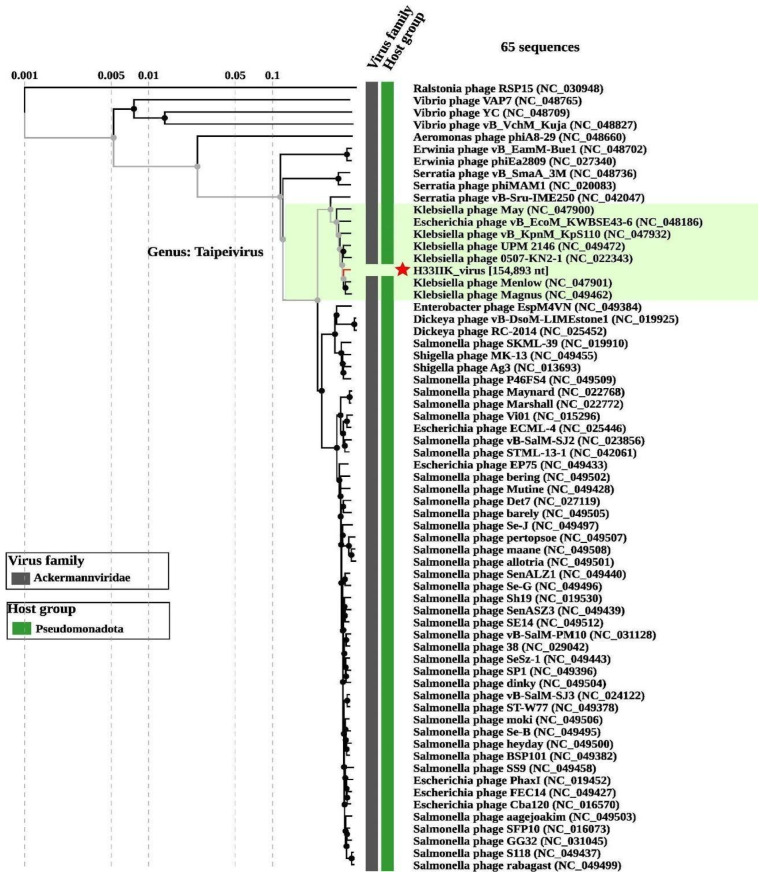
The phylogenetic tree of viral proteins generated by the ViPTree server illustrates the evolutionary relationships among 64 virus sequences. The virus marked with a red star corresponds to the phage isolated from wastewater samples. This phage has been categorized within the *Klebsiella* phage clade, indicating its evolutionary and taxonomic proximity. The tree branches emphasize the diversity and relationships among different phages, while the scale at the top reveals the genetic distance between sequences.

In addition, the bacteriophage H33IIK has been categorized within the *Ackermannviridae* family and the *Taipeivirus* genus, as observed in Figure 6.

**Figure 7:**
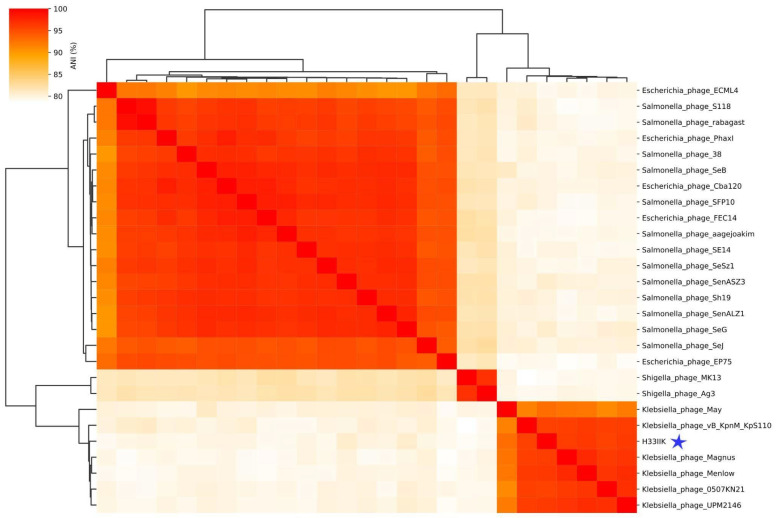
Comparative Average Nucleotide Identity (ANI) analysis of the complete genomes of various phages, including the isolate H33IIK (blue star). The heat map shows the percentage of identity between phages, while the accompanying dendrogram represents phylogenetic relationships based on genomic similarity. Notably, phage H33IIK clusters within the *Klebsiella* phage clade, highlighting its genetic proximity to this specific group.

Furthermore, the evolutionary relatedness between the isolated phage and other members of the same family suggests that they may share similar host adaptation strategies. The Comparative Average Nucleotide Identity (ANI) analysis shown in Figure 7 highlights this genomic similarity, indicating that the phage is closely related to other viruses infecting *Klebsiella* spp., a genus of bacteria recognized for its multidrug resistance and association with nosocomial infections.

To complement the heatmap shown in Figure 7, we extracted the pairwise ANI values between H33IIK and the closest ICTV-classified Taipeivirus members, see Supplementary Materials. H33IIK showed ANI values of 96.36% with *Klebsiella* phage Menlow, 96.18% with *Klebsiella* phage UPM2146, 96.12% with *Klebsiella* phage Magnus, 95.76% with *Klebsiella* phage vB_KpnM_KpS110, and 95.69% with *Klebsiella* phage 0507KN21. All values exceed the ICTV-recommended genus-level threshold of 95%, confirming that H33IIK clusters within the genomic diversity and evolutionary range of the genus Taipeivirus.

**Figure 8:**
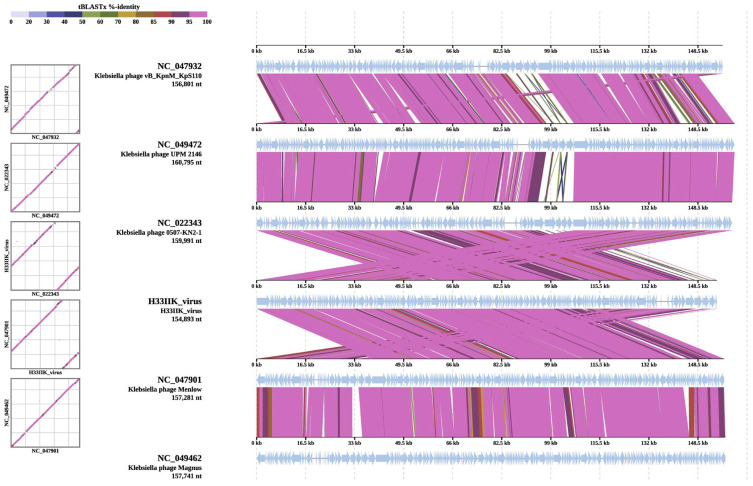
This figure presents BLASTx alignments of the *Klebsiella* phage H33IIK genome, highlighting conserved regions and genomic variations. The color scheme represents sequence similarity: dark purple indicates high conservation, while lighter shades of purple denote genetic divergence, illustrating phage diversity and adaptation. The horizontal axis represents genome length in kilobases (kb), while the vertical axis displays percentage sequence identity, facilitating the assessment of evolutionary and functional relationships among these viral entities. This visualization provides insights into genetic conservation, divergence patterns, and potential functional adaptations within *Klebsiella* phages.

A comparative genomic analysis was conducted, which revealed that *Klebsiella* phage H33IIK shares a high degree of sequence similarity and synteny with other members of the Ackermannviridae family. As demonstrated in Figure 8, the tBLASTx alignment revealed extensive regions of nucleotide identity with *Klebsiella* phages vB_KpnM_KpS110 (NC_047932), UPM 2146 (NC_049472), 0507-KN2-1 (NC_022343), Menlow (NC_047901), and Magnus (NC_049462). The majority of genomic segments exhibited identity values exceeding 80%, as depicted by the predominant pink and purple shades, thereby signifying a conserved genomic organization among these phages. The observed synteny suggests that H33IIK likely shares similar structural and functional gene arrangements with its close relatives, thereby reinforcing its phylogenetic placement within the Ackermannviridae family and its adaptation to *Klebsiella* hosts.

## 3. Discussion

The increasing prevalence of MDR *K. pneumoniae* in hospital settings is a growing public health concern [25]. The global rise in MDR *K. pneumoniae* highlights the urgent need for novel antimicrobial strategies, as existing antibiotics are increasingly ineffective [16,26]. In this context, the present study investigates the potential of bacteriophages [27]. In this regard, the identification of bacteriophages with specific lytic capabilities is highly valuable. Following established protocols [28,29,30,31,32], bacteriophage H33IIK was isolated from wastewater [31], which is a well-established and frequent source for the isolation of phages targeting human pathogens and is frequently overlooked by local health authorities.

The host range of phage H33IIK was found to be highly specific to *K. pneumoniae*. Phage H33IIK exhibited lytic activity against *K. pneumoniae* ATCC BAA-2814 and was capable of lysing 8 out of 50 multidrug-resistant (MDR) *K. pneumoniae* strains. These strains were resistant to extended-spectrum β-lactams and carbapenems, and displayed virulence traits including capsule production and a hypermucoviscous phenotype [33]. It is well established that many bacteriophages encode enzymes capable of degrading capsular polysaccharides, thereby facilitating access to bacterial cell surfaces and enhancing infection efficiency [34]. This mechanism could account for our findings, as all susceptible MDR strains exhibited capsular and hypermucoviscous features [33]. Consequently, it is plausible that H33IIK encodes enzymatic activity or other capsular-targeting proteins that contribute to its lytic efficacy [35]. However, functional characterization of these putative enzymes will be required to confirm this hypothesis.

Furthermore, H33IIK was tested against various members of the Enterobacterales order from different genera; H33IIK exhibited lytic activity exclusively against *K. pneumoniae* strains. This high specificity underscores its potential as a possible targeted therapeutic agent for phage therapy [15,22,36]. Also, other phages with narrow host ranges have demonstrated the ability to co-evolve with their bacterial hosts, adapting to resistance mechanisms over time. However, such specificity also renders them self-limiting, as they may be eliminated in the absence of their host, potentially aiding in the regulation of infection sites [26]. In this study, the strict host specificity of phage H33IIK, restricted to a single bacterial species, suggests that its narrow host range may be determined by strong affinity for specific bacterial receptors, likely mediated by molecular recognition mechanisms such as tail spike proteins or tail fibers [37,38].

Interestingly, members of this class are frequently detected in the human gut microbiome [39], which is consistent with the isolation of H33IIK from untreated wastewater, another complex and diverse microbial niche. The optimal multiplicity of infection (MOI) for H33IIK was determined to be 0.1. Although this value is higher than those reported in some previous studies [16], it reflects the infection dynamics of this phage. The MOI represents the ratio of phages to host cells during a single infection cycle, encompassing adsorption, genome injection, replication, and progeny release, typically under conditions where most bacterial cells are infected by a single virion. This parameter is crucial for determining key growth characteristics, including the latent period and burst size. In the case of H33IIK, an incubation period of 10 min [30], together with a satisfactory burst size, indicates efficient replication kinetics and strong proliferative capacity [16]. Killing curves were performed to evaluate the antibacterial activity of bacteriophage H33IIK against its host, *Klebsiella pneumoniae* ATCC BAA-2814, at different multiplicities of infection (MOIs). The killing curves demonstrated that bacteriophage H33IIK efficiently inhibited the growth of *K. pneumoniae* regardless of the MOI applied (1, 0.1, and 0.01) [40].

Furthermore, phage H33IIK exhibited remarkable tolerance to extreme pH conditions, maintaining stability at both pH 3.0 and 10.0, in agreement with previous observations [41]. This physicochemical resilience contrasts with reports on other *Klebsiella*-infecting phages, which generally display reduced stability under similar conditions [16,42]. The ability of H33IIK to withstand both acidic and alkaline environments underscores its potential utility in biotechnological applications [36,43]. In addition, H33IIK demonstrated notable thermal stability, retaining its titer after incubation at 50 °C for 60 min. Such robustness highlights its potential as a promising candidate for food biocontrol interventions [16]. An evaluation of the effect of 10% chloroform on phage H33IIK revealed no significant sensitivity, suggesting that the viral capsid proteins are resistant to chloroform. It could be explained by the absence of a lipid envelope, as non-enveloped viruses typically rely on robust capsid structures for environmental stability [44].

This lytic phage, a member of the Ackermannviridae family, displayed marked specificity toward multidrug-resistant *K. pneumoniae* strains, making it a promising candidate for the development of biotechnological applications. Phylogenetic analysis placed H33IIK within the Taipeivirus genus, thus grouping it alongside phages such as 0507-KN2-1 and UPM2146, which are known for their efficacy against *Klebsiella* spp. [45]. This phylogenetic relationship suggests a common evolutionary origin, as well as similarities in genomic organization and infection mechanisms. Average nucleotide identity (ANI) analysis corroborated the close relationship between H33IIK and other Klebsiella-infecting phages, especially with 0507-KN2-1. The ANI values obtained for H33IIK (95.69–96.36%) demonstrate a high genomic relatedness to ICTV-classified members of the genus Taipeivirus, supporting its placement within this taxonomic group. These values not only exceed the genus-level cutoff of 95% but also reflect the conserved genomic synteny typically observed among Taipeivirus phages. This high genomic similarity supports its taxonomic classification and allows inferring host compatibility and infectious behavior, factors determining its application in targeted therapies. This approach has been widely adopted in modern phage taxonomy, supported by genomic data to establish precise phylogenetic relationships [46].

Comparative BLASTx alignment revealed highly conserved genomic regions between H33IIK and other phages of the same clade, such as 0507-KN2-1, UPM2146, and Menlow. These regions include structural genes essential for viral assembly, as well as genes involved in bacterial lysis. However, divergent regions were also observed that could reflect specific adaptations of H33IIK, possibly associated with variations in bacterial receptors or immune evasion mechanisms [47]. The circular genetic map analysis revealed a genomic architecture consistent with that of lytic phages with therapeutic potential. The structural genes (capsid, tail), accessory proteins, and lytic enzymes, such as lysins, responsible for the degradation of bacterial peptidoglycan, were identified. These enzymes have been widely studied as antimicrobial tools due to their efficacy and specificity [24]. Furthermore, the H33IIK genome lacks genes associated with bacterial virulence, antibiotic resistance, and genomic integration, such as integrases, reinforcing its safety profile and strictly lytic nature.

Recent studies have documented that phage of the Taipeivirus genus, such as the one reported by [24], exhibit lytic activity without carrying resistance or virulence genes, meeting key criteria for phage therapy applications [48]. The conservation of key functional elements and the absence of risk genes reinforce its clinical applicability. However, additional in vivo studies are required to validate its efficacy, stability, and safety, as well as to explore its combined use in multiphage formulations [49].

## 4. Materials and Methods

### 4.1. Geographic Distribution of the Sample Points

The wastewater sampling points located along the Huatanay River were visualized using QGIS (version 3.40), a free and open-source geographic information system (https://qgis.org/). The political division at the departmental level was obtained from the INEI via the Peruvian government’s Open Data Platform (https://www.datosabiertos.gob.pe/dataset/limites-departamentales/resource/2aa1a2a6-55e6-4090-9152-dc223fdf9930, accessed on 5 October 2024). All spatial data were processed using the WGS 1984 datum (World Geodetic System), Zone 18, with a 6° longitudinal width based on the Universal Transverse Mercator (UTM) projection. Sampling point locations were georeferenced following the recommendations of the National Geographic Institute for the use of official datum and cartographic projection standards in Peru (https://app8.ign.gob.pe/GestionDocumental/Documento.aspx?id=2634, accessed on 5 October 2024, https://www.fao.org/faolex/results/details/es/c/LEX-FAOC064387, accessed on 5 October 2024). Additionally, the 12 sampling points were established based on the six marginal strips defined by the National Water Authority (ANA) in 2013, 2014, and 2015. 72 surface water samples (500 mL each) were collected during November 2022 and February 2023, following Peruvian regulations, specifically Chief Resolution No. 010-2016-ANA and Directorial Resolution No. 2254/2007/DIGESA/SA.

### 4.2. Phage Isolation and Purification

72 samples of surface water were analyzed individually and stored at 4 °C for 24 h to precipitate the suspended solids, then filtered with the help of a vacuum pump to remove the organic matter. For the enrichment culture, the filtrate was used in an 8 mL aliquot in tubes; 1000 µL of *Klebsiella pneumoniae* ATCC BAA 2814 (Microbiologics KWIK STIK Ref: 01263K, St. Cloud, MN, USA) culture in logarithmic phase was added; likewise, 2 mL of 5× TSB (Tryptone Soy Broth, Oxoid Ref: CM0129B, Hampshire, UK) was incorporated, and incubated at 37 °C/24 h, taken as a reference from [28] and modified. For phage isolation, the culture was centrifuged at 3000 *g*/15 min, and the supernatant was filtered through a 0.22 µm syringe (Membrane Solutions Ref: SFNY030022NI, Seattle, WA, USA) into a sterile tube for purification.

### 4.3. The Spot Assay

For isolation of lytic bacteriophages, a culture of *K. pneumoniae* ATCC BAA 2814 in log phase at (ca. 0.4 OD_600_) corresponding to approximately 10^8^ CFU/mL was used. Bacterial culture was performed by plating on TSA (Tryptone Soy Agar) (Oxoid Ref: CM0131R, Hampshire, UK) plates and left at room temperature for 10 min. Then, 10 µL of the phage filtrate (1 drop) was inoculated and left for 10 min at room temperature. The culture was then incubated at 37 °C/24 h to observe the presence of plaques [50].

### 4.4. The Double-Layer Agar

For the isolation of bacteriophages for *K. pneumoniae* BAA ATCC 2814 (Microbiologics KWIK STIK Ref: 01263K), plates that exhibited clear plaques were selected by the drip method. Phage-containing filtrates showing lytic potential were serially diluted (10^−1^ to 10^−7^) in 1000 μL of 0.85% saline solution. To each dilution, 1000 µL of a *K. pneumoniae* BAA ATCC 2814 (Microbiologics KWIK STIK Ref: 01263K) culture (ca. 0.4 OD_600_) was added, followed by incubation at 37 °C for 15 min. Subsequently, a TSB (Oxoid Ref: CM0129B, Hampshire, UK) with 0.7% Bacto agar (Oxoid Ref: LP0011B, Hampshire, UK) was added at 45 °C, homogenized, and incorporated into Petri dishes with previously prepared TSA (Oxoid Ref: CM0131R, Hampshire, UK). These were incubated at 37 °C/24 h to isolate and quantify the lysis plaques [29]. Finally, the lysis plaques were cut, and they were placed in microcentrifuge tubes with 500 μL of 0.85% saline solution and centrifuged at 5000× *g*/10 min. The supernatant was filtered with 0.22 µm syringe filters and stored at 4 °C. This procedure was performed three times [51].

### 4.5. Amplification and Titration of Bacteriophages

To increase the viral concentration, 10 mL of TSB (Oxoid Ref: CM0129B, Hampshire, UK) was inoculated with 1 mL of bacteriophage suspension and 1 mL of bacterial suspension of *K. pneumoniae* ATCC BAA-2814 (Microbiologics KWIK STIK Ref: 01263K) culture in the logarithmic growth phase (ca. 0.4 OD_600_). The culture was incubated at 37 °C/24 h to monitor the clearance of the culture medium compared with the control culture, confirming the presence of lytic bacteriophages. After incubation, the suspension was centrifuged at 3000× *g*/10 min. at 4 °C, and the supernatant was filtered through a 0.22 µm syringe filter (Membrane Solutions Ref: SFNY030022NI, Seattle, WA, USA). The filtrate was then stored at 4 °C, following a modified protocol described by [52].

The phage titer (PFU/mL) of the final filtrate was determined using the double-layer agar method and calculated according to the formula described in [53].

### 4.6. Multidrug-Resistant (MDR) K. pneumoniae Strains

The MDR *K. pneumoniae* strains were clinical isolates previously characterized and described in [33]. These isolates were subsequently used to determine the host range of the phage.

### 4.7. Host Range Determination

To evaluate the host range of the bacteriophage H33IIK for *K. pneumoniae* ATCC BAA 2814 (Microbiologics KWIK STIK Ref: 01263K), the lytic capacity was tested against 50 strains of MDR *K. pneumoniae*, as well as four strains each of *Enterobacter cloacae*, *Proteus* spp., *E. coli*, and *Pseudomonas aeruginosa* resistant to β-lactam antibiotics, using the spot assay in triplicate [30].

### 4.8. Determination of Optimal Multiplicity of Infection (MOI)

A volume of 100 µL of phage suspension at a concentration of 10^5^ PFU/mL was mixed with 100 µL of *K. pneumoniae* ATCC BAA-2814 (Microbiologics KWIK STIK Ref: 01263K) in phase log OD_600_ (0.7) equivalent to 10^8^ CFU/mL. They, with varying concentrations of 10^4^, 10^5^, 10^6^, 10^7^, and 10^8^ PFU/mL (bacterial suspension). Each mixture was added to 5 mL of Luria–Bertani broth (Merck, Darmstadt, Germany) and incubated at 37 °C with shaking for 6 h. Following incubation, the suspensions were centrifuged at 6000 *g*/15 min. and the supernatants were filtered through 0.22 µm syringe filters (Membrane Solutions Ref: SFNY030022NI, Seattle, WA, USA). The resulting phage titers were quantified using the double-layer agar method in triplicate. The bacteria-to-phage ratio that yielded the highest phage titer after 6 h was considered the optimal multiplicity of infection (MOI), as described by [30].

### 4.9. One-Step Growth Curve of Bacteriophage

A culture of *K. pneumoniae* ATCC BAA-2814 (Microbiologics KWIK STIK Ref: 01263K) grown in Luria–Bertani (LB) medium supplemented with 2 mM CaCl_2_ (Merck Ref: 1.02378, Darmstadt, Germany), was used at the logarithmic phase (ca. OD_650_ 0.5). The bacteriophage suspension (3.2 × 10^8^ PFU/mL), marking this as time zero. The culture was incubated for 5 min to allow phage adsorption, after which serial dilutions were performed following the method described by [54]. Aliquots were taken every two minutes and plated using the double-layer method to determine the phage titer. The obtained data were normalized by multiplying the values of flask A and absorption control by 10, flask B by 100, and flask C by 1000 [54].

### 4.10. Killing Curve

A mid-logarithmic phase culture of *K. pneumoniae* ATCC BAA-2814 (Microbiologics KWIK STIK Ref: 01263K) (OD_600_ 0.524) was inoculated with bacteriophage H33IIK at different multiplicities of infection (MOI: 1, 0.1, and 0.001) in flasks containing 100 mL of LB broth and incubated at 37 °C without agitation. Samples of 1 mL were collected every 30 min for a total period of 270 min. Bacterial growth was monitored by measuring the optical density at 600 nm using a spectrophotometer (Eppendorf AG Ref: 2233, Hamburg, Germany). A phage-free culture was included as the control [40].

### 4.11. Thermal Stability and pH

For thermal-stability testing, 100 μL bacteriophage lysate (10^8^ PFU/mL) was mixed with 900 μL SM buffer. Samples were maintained in a water bath ranging from 20 °C to 90 °C for 60 min. For pH-stability testing, 100 μL of bacteriophage sample (10^8^ PFU/mL) was mixed in a tube containing SM buffer with a pH ranging from 2 to 13 (adjusted using NaOH or HCl) and incubated for 1 h at 37 °C. The phage titer was determined by the double-layer agar method, as previously described by [55]. All experiments were conducted in triplicate.

### 4.12. Sensitivity to Chloroform

Chloroform (Fluka Ref 25690, Bruchs, Switzerland) was added to the phage suspension (10^8^ PFU/mL in SM buffer) to achieve a final concentration of 10% (*v*/*v*). It was incubated with shaking at room temperature for one hour. To determine the phage titer, the cultures were carried out using the double method in triplicate [55].

### 4.13. Genome Sequencing and Bioinformatic Analysis

Phage DNA extraction was performed using Viral Nucleic Acid Extraction Kit II, cat. VR300 (Geneaid, New Taipei City, Taiwan). The library was prepared with the Nextera XT DNA Library Prep Kit. The sequencing described in this study was performed on Illumina^®^ MiSeq i100 Plus with read length of 151 bp, paired-end; the base calling accuracy was measured by Phred Quality Score (Q-score) of 30%. The reads obtained from the sequencing were visualized with the fastQC program [56] (http://www.bioinformatics.babraham.ac.uk/projects/fastqc/, accessed on 20 March 2025) to filter sequences of good quality to obtain reliable results, and then the adapters were removed with Trimomatic [57]. Then they were assembled under the de novo assembly strategy using Spades V3.15.3 [58] with the viralSpades option. The integrity of the assembly was evaluated using QUAST V 5.2.0 [59], the preliminary identification of the contigs that contained phage sequences was carried out with PhageClouds (http://phagecompass.dk/, accessed on 20 March 2025) and with Kraken [60] against the viral database, for a manual separation of those contigs that have virus sequences. For annotation, selected contigs were annotated using the RAST web server interface [61] and the Pharokka program [62]. Finally, the genomic map was designed with Proksee [63].

### 4.14. Life Cycle Prediction, Resistance, and Virulence Genes

The PhageScope server [64] was employed to predict the phage lifestyle and assess the presence of lysogeny-related elements. In addition, AMRFinderPlus v4.0.3 [65] and ResFinder v4.7.2 [66] were used to identify antimicrobial resistance genes, while the VirulenceFinder platform [67] was applied to detect potential virulence factors. Default parameters were used in all analyses, with a minimum identity threshold of 90% and a minimum coverage of 60%.

### 4.15. Therapeutic Suitability Prediction and Phylogenomic Analysis

For the prediction of therapeutic suitability, we used PhageLeads [68]. The Viral Proteomic Tree (VIPTree) server was used to create a proteomic tree [69], restricted to prokaryotic phage genomes. Finally, the phage sequences were compared with other genome sequences present in the NCBI database using the BLAST tool v 2.17.0 [70]. Then, genomes of similar phages of the genus *Taipeivirus* were downloaded to calculate the average nucleotide identity (ANI).

## 5. Conclusions

The phenotypic and genomic characterization of bacteriophage H33IIK identifies it as a strictly lytic *Taipeivirus* infecting *Klebsiella pneumoniae*. Phenotypically, H33IIK exhibits a narrow host range, efficient infection kinetics with an optimal MOI of 0.1, a short latent period, a high burst size, and strong lytic activity across a range of MOIs. The phage shows notable physicochemical stability over a broad range of temperatures and pH values and resistance to chloroform. Genomic analysis reveals a modular genome typical of *Ackermannviridae*, encoding structural, replication, and lysis-related proteins, and lacking genes associated with lysogeny, virulence, or antimicrobial resistance. Together, these data provide a concise biological profile of H33IIK and expand current knowledge of *Taipeivirus* phage diversity and virus–host interactions in *K. pneumoniae*.

Future research should prioritize in vivo efficacy and safety studies of phage H33IIK in relevant animal models to support its biotechnological development. Its incorporation into phage cocktails could broaden coverage against diverse MDR *K. pneumoniae*.

## Figures and Tables

**Table 1:** Lytic activity of phage H33IIK against clinical strains of MDR *K*. *pneumoniae*.

Bacterial Code	Strain	Type of Resistance	Resistance Genes	Antibiotic Resistance		Phage H33IIK	
				[CRO]	[CAZ]	Lytic Activity	Titer
HR86	*K. pneumoniae* subsp. *pneomoniae*	ESBL+	bla CTX-M, bla TEM, bla SHV	R	R	(+)	3.0 × 10^7^ PFU/mL
HR187	*K. pneumoniae* subsp. *ozonae*	ESBL+	bla SHV	R	R	(+)	3.0 × 10^7^ PFU/mL
HR207	*K. pneumoniae* subsp. *pneumoniae*	ESBL+	bla CTX-M, bla TEM, bla SHV	R	I	(+)	3.0 × 10^7^ PFU/mL
ES192	*K. pneumoniae* subsp. *pneumoniae*	ESBL− Carbapenemase	bla KPC, bla CTX-M, bla TEM, bla SHV	R	R	(+)	3.0 × 10^7^ PFU/mL
HAL91	*K. pneumoniae* subsp. *pneumoniae*	ESBL− Carbapenemase	bla KPC, bla CTX-M, bla TEM, bla SHV	R	R	(+)	3.0 × 10^7^ PFU/mL
HR189	*K. pneumoniae* subsp. *pneumoniae*	ESBL+	bla SHV	R	R	(+)	3.0 × 10^7^ PFU/mL
HR198	*K. pneumoniae* subsp. *pneumoniae*	ESBL+	bla SHV	R	R	(+)	3.0 × 10^7^ PFU/mL

Note: The susceptibility profile (I: intermediate; R: resistant), and the susceptibility discs used (ceftriaxone [CRO] and ceftazidime [CAZ]) were denoted accordingly. Molecular screening revealed the presence of β-lactamase genes among the isolates (blaKPC, blaNDM, blaIMP, blaVIM, blaCTX-M, blaTEM, and blaSHV).

**Table 2:** Non-Klebsiella strains tested for host range.

Code	Strain	Bacterial OD_600_	Type of Resistance	Bacteriophage Titer	Lytic Activity
HAL114	*Enterobacter cloacae*	0.5	Carbapanemase	3.0 × 10^7^ PFU/mL	ND
ES355	*Escherichia coli*	0.5	ESBL+	3.0 × 10^7^ PFU/mL	ND
HAL15	*Pseudomonas aeruginosa*	0.4	Carbapanemase	3.0 × 10^7^ PFU/mL	ND
ES269	*Proteus* spp.	0.4	Carbapanemase	3.0 × 10^7^ PFU/mL	ND

Note: ND: No lytic activity of phage H33IIK detected.

## Data Availability

Illumina raw reads of isolated have been submitted to NCBI under BioProject PRJNA1129429, with SRA Experiment accession SRX26553861 and Run accession SRR31170557. Complementary data will be made available on request.

## Supplementary Materials

The following supporting information can be downloaded at: https://www.mdpi.com/article/10.3390/antibiotics15040365/s1, Table S1: The optimal multiplicity of infection (MOI) of H33IIK. Table S2: Data from an OSG experiment. Table S3: Data of killing curve of *K. pneumoniae* ATCC BAA 2814 using the phage H33IIK. Table S4: Temperature tolerance of phage H33IIK. Table S5: pH sensitivity of phage H33IIK. Table S6: Chloroform sensitivity of phage H33IIK. Table S7: ANI cluster map matrix.

## Abbreviations

The following abbreviations are used in this manuscript:

MDR	Multidrug-resistant
GNB	Gram-negative bacteria
ATCC	American Type Culture Collection
